# Cassava Waste Starch as a Source of Bioplastics: Development of a Polymeric Film with Antimicrobial Properties

**DOI:** 10.3390/foods14010113

**Published:** 2025-01-03

**Authors:** Yuri D. O. Silveira, Adriana S. Franca, Leandro S. Oliveira

**Affiliations:** 1 Programa de Pós-Graduação em Ciência de Alimentos (PPGCA), Universidade Federal de Minas Gerais, Av. Antônio Carlos, 6627, Belo Horizonte 31270-901, MG, Brazil; yuridvcp@gmail.com (Y.D.O.S.); leandro@demec.ufmg.br (L.S.O.); 2 Departamento de Engenharia Mecânica (DEMEC), Universidade Federal de Minas Gerais, Av. Antônio Carlos, 6627, Belo Horizonte 31270-901, MG, Brazil

**Keywords:** bioplastics, cassava waste, polysaccharides, starch

## Abstract

Polysaccharides represent the most abundant biopolymers in agri-food wastes and thus are the most studied polymers to produce biodegradable films for use in packaging. Starch is among the major polysaccharides extracted from food and agricultural waste that have been used as precursor material for film production. Therefore, the present study aimed at producing an active film with antimicrobial properties using starch extracted from cassava waste and oil extracted from cloves. The antimicrobial activity of the produced films was tested against *Staphylococcus aureus*, *Salmonella* Typhimurium and *Listeria monocytogenes*. Cassava periderm and cortex were bleached with either NaClO or H_2_O_2_ before starch aqueous extraction. The active films’ antimicrobial effectiveness was assessed by the formation of inhibitory halos around film disc samples in an agar diffusion method. The inhibition zone diameters were statistically similar for all microorganisms, with an average diameter of 11.87 ± 1.62 mm. The films presented an average water vapor permeability of 0.14 g mm/m^2^ h kPa, an average tensile strength of 0.17 MPa and an elongation at break of 32.90%. Based on the determined properties, the produced films were deemed adequate for use in food packaging, in which antimicrobial activity is paramount.

## 1. Introduction

Polymeric materials are significantly relevant for food packaging, and the need to eliminate environmental issues arising from their disposal has stimulated studies on biodegradable packaging materials [1]. Among the possibilities, many studies have focused on polysaccharides that are abundant in agricultural products, including starch, given its availability in a wide variety of plants as well as its thermoplastic capacity and film-forming ability [2,3]. The main sources of starch worldwide are corn (65%), sweet potato (13%) and cassava (11%), with the latter being of economic interest in several regions around the world, including South America, Asia and Africa [3].

Cassava starch has been extensively studied for the development of plastic packaging materials [3,4], and recent works have focused on: (i) blending starch with other polymeric materials, either natural [5] or synthetic [6]; (ii) the addition of reinforcing materials [7]; and (iii) the addition of polyphenols and essential oils to increase antioxidant and antimicrobial activities [7,8]. However, the number of studies that employ starch from cassava residues or by-products is still small [3]. These residues include cassava peel, the main waste from the peeling of cassava tuber, that accounts for over 5% of the cassava root mass [9] and is discarded in the production of either cassava flour or starch.

Tafa and collaborators [10] prepared films employing starch extracted from cassava peel and reinforced with silica nanoparticles (SNP) extracted from rice husks and bamboo leaves. Glycerol was used as plasticizer, and essential oil from rosemary leaves (REO) was also added. The addition of SNP led to increases in tensile strength and elongation at break. However, as SNP concentrations increased over a certain value, mechanical properties were weakened, probably due to the formation of agglomerates, impairing interaction between the filler and polymeric matrix. The films with added REO showed some inhibition activity against food poisoning bacteria (*S. aureus* and *E. coli)*, even though the inhibition zone was relatively small in comparison to the reference (gentamycin).

A recent study [9] proposed the use of enzymatic hydrolysis to obtain polymeric films from cassava peels. A blend of cellulase, xylanase and glucanase was used for the enzymatic treatment. Although the use of enzymes provided a decrease in film strength, flexibility increased, probably because some of the small carbohydrate units acted as plasticizers, increasing elasticity and reducing film stiffness. Cassava peel was also recently used as a source of both starch and cellulose nanofibers to prepare a polymeric film blended with locust bean galactomannans [1]. The addition of the galactomannans decreased film strength but provided a slight improvement in flexibility. Even though the added nanofibers did not improve the film’s mechanical properties, their vapor barrier was enhanced. Although cassava-based starch has been extensively studied as a potential material for biodegradable food packages [3], some drawbacks still remain, including its hydrophilic nature, high moisture absorption and high susceptibility to microorganisms [10]. The possibility of microbial growth can lead to a significant reduction in the lifetime of films and consequent shelf life of packed products, being one of the biggest challenges of the food packaging industry [10].

It is noteworthy to point out that most of the published articles on active films based on cassava starch employ starch extracted from the cassava pulp, which is an actual food product and not a waste. Only a few articles have dealt with cassava peel [1,9,10] and have either used solely the cortex portion of the peel, leaving the periderm as waste [9,10], or focused on starch/galactomannan blends with added nanofibers [1].

In view of the aforementioned, the goal of the present study was to develop polymeric films based on cassava peel starch and to verify the effect of adding clove essential oil (eugenol) on antimicrobial properties. The prepared films were characterized in terms of their physical, structural, mechanical and antimicrobial properties. It is noteworthy to mention that eugenol is classified as “generally recognized as safe” (GRAS) in food products, with well-established pharmacological properties, including antimicrobial, anti-inflammatory, analgesic, antioxidant and anticancer activities, among others [11].

## 2. Material and Methods

### 2.1. Material

Cassava tuber peels were acquired from the local market. The peels, consisting of an external brown corky periderm and an internal off-white to pinkish cortex, were washed in tap water, pat dried and stored in plastic bags at −20 °C for further use. Prior to starch extraction, the stored peels were thawed and separated into two groups: one in which the brown corky periderm was removed and discarded, herein termed NCP; and another in which the corky periderm was not removed, with the sample termed WCP. Clove buds were acquired at a local market in Belo Horizonte, Brazil. The following microorganisms were kindly donated by FIOCRUZ–Instituto René Rachou (Belo Horizonte, Brazil): *Staphylococcus aureus* CCCB S007, *Salmonella enterica* Typhimurium (CCCB S004) and *Listeria monocytogenes* serotype 4 b (ATCC 19115).

### 2.2. Methods

#### 2.2.1. Starch Extraction

Both groups of samples, i.e., NCP and WCP, were separately processed in an industrial blender (Metvisa Bing, Brusque, Brasil) together with 1 L of distilled water until smooth pastes were formed (about 5 min each). These were then permeated through a polypropylene mesh, with the filtered solution predominantly consisting of starch. The retentate was again processed in the industrial blender and subsequently filtered. The filtered solution of the second processing cycle was combined with that of the first cycle. The starch solution was taken to a convective oven at 50 ± 2 °C for 12 h to evaporate the water. Following the dehydration of the solution, the dried extracts were macerated in a porcelain mortar and classified through a Tyler 42 mesh sieve, and the powder passing through the sieve (D < 0.355 mm) was employed for film preparation. These powders were analyzed for their polysaccharide contents, and starch was the predominant one, with average contents of 72.30 ± 0.57 g/100 g and 68.9 ± 0.43 g/100 g for NCP and WCP, respectively. The remainder of the extract was mostly comprised of hemicellulose and cellulose (25.6 ± 0.49 g/100 g).

#### 2.2.2. Oxidative Treatment of the Dry Extract

Ten grams of the dried extracts were oxidized with a solution of NaClO or a H_2_O_2_ solution, both at three distinct concentrations, 2, 6 and 10%, resulting in a bleached powder. The bleaching process consisted of stirring the powder with the NaClO bleach solution at room temperature for 1 h, with pH adjusted to 8.4 ± 0.5 by adding a 3% NaOH sodium hydroxide solution. After 1 h, the pH was decreased to 6.8 by adding a 5% H_2_SO_4_ solution, followed by the addition of 0.44 g of sodium bisulfite to remove excess NaClO. The treatment with H_2_O_2_ was carried out by stirring the solution for 3 h, followed by the addition of 0.44 g of sodium bisulfite to remove excess H_2_O_2_. Following both bleaching processes, the extracts were dried in a convective oven at 45 ± 2 °C for 12 h and subsequently macerated in a porcelain mortar. The obtained powders were used for the preparation of the polymeric films.

#### 2.2.3. Extraction of Clove Buds’ Essential Oil

Clove buds’ essential oil, the potential antimicrobial agent, was prepared by hydro distillation. Thirty grams of dried clove buds were finely powdered and subsequently inserted into a filter paper extraction thimble and placed into a 500 mL reflux flask connected to a Soxhlet apparatus. The buds were extracted with distilled water at 100 °C for 5 h. Following the hydro distillation process, the collected distillate (i.e., aqueous extract) was placed in a separation funnel to which chloroform was added. The funnel was thoroughly agitated, and the separated organic phase collected and dehydrated with anhydrous sodium sulfate. The dehydrated organic extract was then rotary evaporated until all the chloroform was removed and the essential oil concentrated. The essential oil was stored in a refrigerator until further use. The oil composition was eugenol (84.8–87.1%); eugenol acetate (2.9–3.5%); β-caryophyllene (0.92–1.6%); and α-humulene (0.41–0.67%).

#### 2.2.4. Film Preparation

The unbleached and bleached extracts followed the same procedure for the preparation of the starch films. To prepare the filmogenic solution, 2 g of the dried extracts and 0.6 g of glycerol were added to 37.4 mL of distilled water, thus comprising 40 g of filmogenic solution. Clove buds’ essential oil (0.25, 0.5, 1.0 and 2.0 g) was also added. The filmogenic solutions were stirred for 45 min at room temperature and then heated until a temperature of 70 °C was achieved. Subsequently, the filmogenic solutions were transferred to circular polypropylene molds, which in turn were placed in a convective oven at 40 ± 2 °C for 24 h to evaporate all the water. The prepared films were termed NCPF (no corky periderm) and WCPF (with corky periderm).

#### 2.2.5. Film Characterization

Film color determination was carried out with a tristimulus colorimeter (Colorflex, Hunter Associates Laboratory, Reston, VA, USA) using the CIELab system. CIELab is a color space that describes colors using the following values: L*, representing color lightness or how bright or dark a color is; a*, representing the red–green axis, with negative values associated with green and positive values associated with red; and b*, representing the yellow–blue axis, with negative values associated with blue and positive values associated with yellow. The color parameters L*, a* and b* were determined and employed in the calculation of the parameters chroma (c*) and hue value (h*), representing color saturation and tone, respectively. A Fourier Transform Infrared spectrophotometer (IRAffinity-1, Shimadzu, Japan) was used to obtain the FTIR spectra of the produced films. The spectrophotometer was equipped with an attenuated total reflectance (ATR) cell with ZnSe crystal. The spectra were obtained in the wavenumber range from 4000 to 400 cm^−1^, with 20 scans.

The films’ mechanical properties, tensile strength (TS) and elongation at break (EB) were determined in a texture analyzer (TAXTPLUS, Stable Micro Systems, Bruker AXS GmbH, Karlsruhe, Germany) equipped with a 20 N load cell, according to the ASTM standard method D882-18 [12] with minor modifications. Three samples of each film (100 × 25 mm strips) were used for each analysis. The initial grip separation was 50 mm, and the crosshead speed set at 12.5 mm min^−1^. Each film sample was preconditioned for 48 h at 26.6 °C and 44% relative humidity prior to analysis. For the tensile strength calculation, films’ thicknesses were measured with a Mitutoyo micrometer (No 103 137) at five distinct randomly selected locations on each specimen.

Water vapor permeability was determined according to the standard protocol ASTM E96/E96M-24a with slight modifications [13]. The film samples were fixed in air-tight circular capsules with waterproof walls containing 5 g of dry granulated calcium chloride. The system was weighed and placed in a desiccator (21 °C, 75% relative humidity) containing a saturated solution of calcium chloride. The system was weighed every 30 min for five hours. The determination of the water vapor permeability was carried out in quadruplicate, with two repetitions for each film sample. The films’ water vapor permeability, WVP (g/m^2^ s Pa), was calculated by

(1) $$WVP=\left(\frac{w}{t}\right)\left(\frac{e}{A∆P}\right)$$

where *w*/*t* is the slope experimental points of weight gain (g) of the capsules as a function of time, *e* is the film thickness, *A* is the film’s exposed area and Δ*P* corresponds to the pressure differential of water vapor between the film and pure water at 25 °C.

#### 2.2.6. Antimicrobial Tests

The antimicrobial activities of the prepared cassava waste starch films functionalized with clove buds’ essential oil were assessed against *Staphylococcus aureus* CCCB S007, *Salmonella enterica* Typhimurium (CCCB S004) and *Listeria monocytogenes* serotype 4 b (ATCC 19115) by the agar diffusion method. The antimicrobial activity of the essential-oil-incorporated films was determined by cutting the samples into circular shapes of 5.38 mm diameter, subjecting them to ultraviolet radiation for 30 min and placing them on microbially inoculated agar medium surfaces in Petri dishes. The inoculated Petri dishes were priorly incubated for 24 h at 37 °C. The essential-oil-incorporated films’ antimicrobial effectiveness was assessed by the formation of an inhibitory halo around the disc samples. The diameter of the inhibitory halo was measured with a caliper. Since the diameter of the inhibition halos were not uniform surrounding the discs, an average of three measurements for every halo was used to calculate the diameter of the inhibition zone. The effect of essential oil dosage (0.25, 0.5, 1.0 and 2.0 g) was evaluated in preliminary tests against *Staphylococcus aureus* CCCB S007. The preliminary tests demonstrated that the higher the content of clove oil in the films the stronger the antimicrobial activity against *Staphylococcus aureus*. Hence, subsequent studies were conducted at films’ clove oil content of 2 g. Control films, consisting of 5.3 mm discs of filter paper containing 0.49 g of either eugenol or clove essential oil, were also tested for antimicrobial activity against the same micro-organisms. Cassava waste films without antibacterial compounds were tested as well but presented no antimicrobial activity, with the entire cultures being overcrowded with the previously mentioned microorganisms.

## 3. Results and Discussion

### 3.1. Film Characterization

#### 3.1.1. Color

Cassava peels are usually dark colored, and films with such hue are undesirable for food packaging, since consumers tend to select packages that allow them to verify the visual aspects of the product. To overcome this limitation, the peels were bleached with the oxidizing agents NaClO and H_2_O_2_. Oxidized starch is recommended for applications in biodegradable materials, since oxidation promotes improvements in the mechanical and barrier properties of the produced films [14]. Structural modifications imparted on the starch are responsible for the improvements in such properties. NaClO acts by breaking the bonds in the glucose units comprising the starch chain and oxidizing the hydroxyl groups linked to carbon C6 or the diols in carbons C2 and C3, introducing carbonyl and carboxyl groups in those positions. The introduction of such groups into the starch molecules promoted the aforementioned changes in the film properties and imparted a hydrophobic character to the prepared films [14,15].

The cassava waste extracts herein obtained, subjected to bleaching processes with NaClO and H_2_O_2_, became significantly lighter, allowing the resulting films to present a suitable appearance for applications in food packaging. All the resulting films could be easily peeled from the casting tray and were flexible and easily folded. The color parameters determined for the produced films are presented in Table 1. The color of the films approximately matched the colors of the treated precursor materials. The color tone tends to orange, given the hue angle values (~45–80°), with yellowness increasing with the angle. Notice that the treatments of precursor materials with NaClO and H_2_O_2_ led to films that were lighter (higher luminosity values) than those produced from precursor materials without bleaching. Furthermore, the films produced from the peels without the brown corky periderm (NCPF) were lighter than those produced from the peels with the brown corky periderm (WCPF). Interestingly, the treatment of both types of films with NaClO led to films with lighter colors when the concentration of the oxidizing agent was 6%. Upon increasing the NaClO concentration to 10%, the film produced from the resulting precursor material became darker than the film produced from the precursor material treated with NaClO 6%. It seems that, at concentrations higher than 6%, the oxidizing agent generated colored products as it reacted with certain compounds in the precursor material, most likely polyphenols, resulting in colored quinones [16]. Bleaching with H_2_O_2_ was only effective for the precursor material without the brown corky periderm, leading to films that were lighter than all the other films. However, the mass yields of the H_2_O_2_-treated materials were slightly lower than those of the precursor materials treated with NaClO.

Overall, the films produced from the precursor materials treated with 6% NaClO presented more desirable colors (higher luminosity and hue angles) than those produced from the precursor materials treated with NaClO at other concentrations or with H_2_O_2_. Hence, the subsequent studies were carried out with the films from precursor materials treated with 6% NaClO.

#### 3.1.2. Fourier Transform Infrared (FTIR)

**Figure 1:**
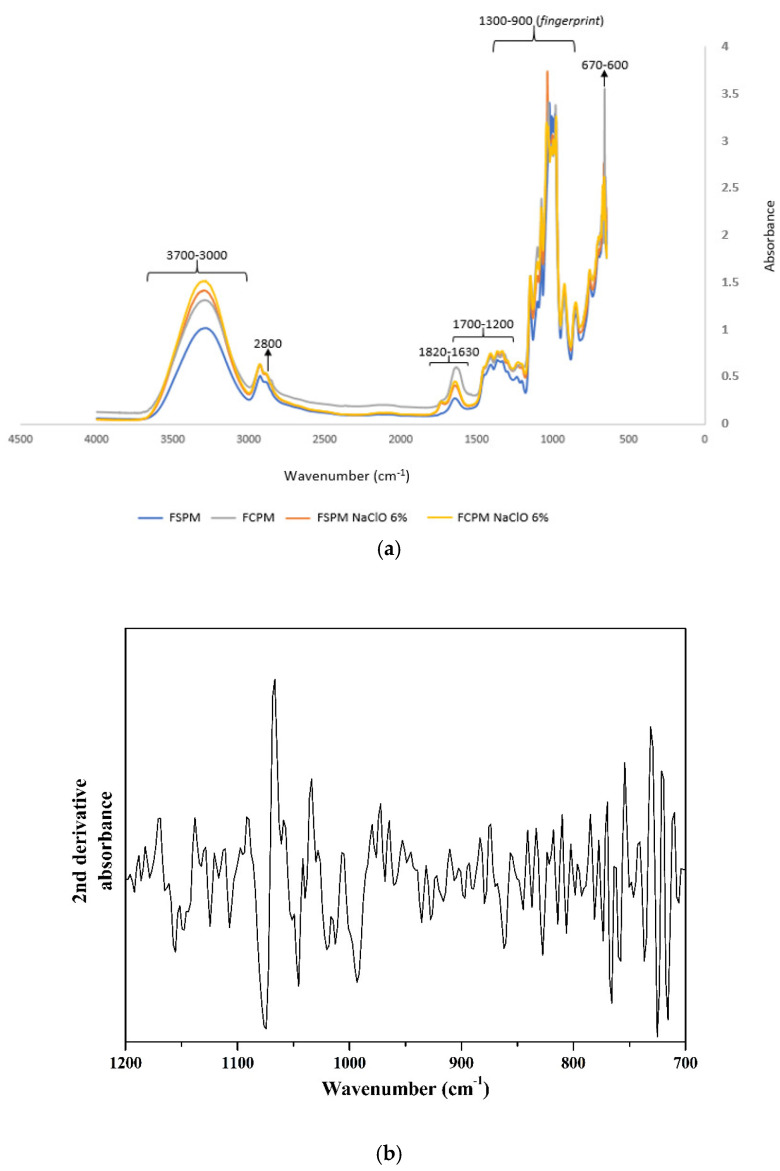
(**a**) FTIR spectra of the films: NCPF (blue line), WCPF (grey line), NCPF 6% NaClO (orange line) and WCPF 6% NaClO (yellow line); (**b**) typical second derivative of the spectra for the prepared films. NCPF: film without corky periderm; WCPF: film with corky periderm.

The FTIR spectra for the films prepared from the untreated precursor materials and from the 6% NaClO-treated precursor materials are presented in Figure 1.

All samples presented similar spectra, without significant differences among them aside from variations in peak intensity in the 3500–3000 cm^−1^ region. The broad band at 3500–3000 cm^−1^ is attributed to the stretching vibration of hydroxyl groups which are intermolecularly and intramolecularly connected. The strong absorption in this region is partially attributed to the higher content of amylopectin in comparison to amylose in the starch from cassava waste [17]. The peak identified at 1647 cm^−1^ is attributed to the bending vibration of water molecules adsorbed into the amorphous portion of the starch. A cluster of unresolved peaks can be observed at the 1400 to 750 cm^−1^ range. This range of wavenumbers encompasses the local symmetry region from 1400 to 1200 cm^−1^, the polysaccharide fingerprint region from 1200 to 800 cm^−1^ and the anomeric region from 970 to 800 cm^−1^, the latter being the region where α and β configurations of the polysaccharides anomeric carbons can be discriminated [18]. The second derivative of the spectrum was taken to obtain a more detailed analysis of the fingerprint region. In the second derivative spectra, three distinctive bands were observed at 1048, 1018 and 996 cm^−1^, respectively attributed to the ordered crystalline structure, absorption by stretching modes in amorphous starch and bonding in hydrated carbohydrate helices [19]. The predominance of starch amorphous structures in the films is evidenced by the high intensity of the bands at 1018 cm^−1^ in all spectra. The band at 927 cm^−1^ is ascribed to the vibration of α–1–4 glycosidic linkages of amylose. Bands at 1148 and 1078 cm^−1^ are ascribed to an anhydro glucose ring C–O stretch of C–O–H in starch and a C–O–C antisymmetric bridge, respectively.

Notice that there are several distinct bands in the region ranging from 1800 to 1200 cm^−1^ and that these bands are more prominent in the spectra for the films based on the precursor material with cassava periderm. It can thus be inferred that phenolic compounds, which are abundantly present in the periderm, are the major contributors to infrared absorption in this region. The band at 1692 cm^−1^ is ascribed to C–O stretching vibration in phenolic compounds, and the band at 1296 cm^−1^ is attributed to the rocking vibration of C–H groups in phenyl rings. The bands at 1180 and 1114 cm^−1^ are respectively attributed to the stretching vibration of the C–OH group and to the phenyl ring bending vibrations in phenolics and their derivatives. The band at 1602 cm^−1^ is due to the stretching vibration of phenyl rings, and that at 1627 cm^−1^ is assigned to the stretching vibration of the C=C ethylenic group in phenyl rings.

#### 3.1.3. Antimicrobial Activity of Functionalized Films

The films functionalized with 2 g clove buds’ essential oil were subjected to antimicrobial tests against *Staphylococcus aureus* and *Listeria monocytogenes* serotype 4B (Gram-positives), as well as against *Salmonella enterica* serovar Typhimurium (Gram-negative). Measurements obtained for inhibition zone sizes are displayed in Table 2.

**Figure 2:**
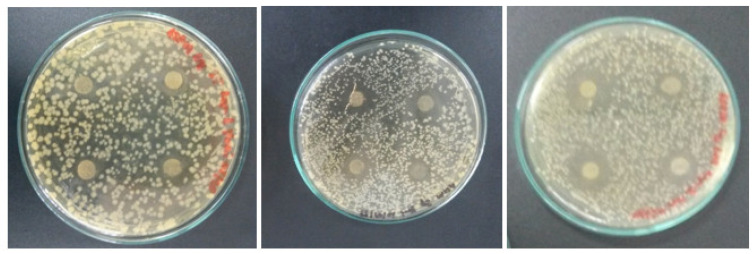
Antimicrobial activity of NCPF against *S. typhimurium* (**left**), *S. aureus* (**middle**) and *L. monocytogenes* (**right**).

All bacteria species were sensitive to the presence of clove oil in the films, and inhibition zones were observed for all the tested films, with a few examples shown in Figure 2. Overall, microbial growth inhibition was more effective for the NCPF film than for the WCPF one (Table 2). Furthermore, the control samples presented higher antimicrobial activities than the prepared film samples. Based on the inhibition zone measurements for the control samples, it can be inferred that the incorporation of essential oil into the filmogenic solution leads to an imprisonment of its antimicrobial agents within the molecular network comprising the formed film, hindering their diffusion through the film and subsequently in the agar medium. Eugenol can interact with the polysaccharide chains in the film matrix via hydrogen bonds, which will hinder its release kinetics and hence its antimicrobial efficiency. In the case of the tests with *L. monocytogenes*, the control samples, both the one with eugenol and the other with clove essential oil, completely inhibited the bacterial growth.

Although it has been strongly hypothesized and at times verified that Gram-positive bacteria are more susceptible than Gram-negative bacteria to the antimicrobial effects of hydrophobic compounds (such as eugenol) [20], no differences in growth inhibition efficacy were observed for Gram-positive and Gram-negative bacteria in the present study. The inhibitory activity of bacterial growth by clove essential oil has been attributed to its major constituents, eugenol and isoeugenol (70–90%), eugenyl acetate (2–17%) and β-caryophyllene (5–12%), with the mechanism of action described as the promotion of increased cell membrane passive permeability and fluidity, followed by the leakage of intracellular components, such as ATP, proteins, nucleic acids and electrolytes, ultimately leading to bacterial cell death [20,21]. The interaction of these hydrophobic compounds with the cell membrane causes its destabilization with a consequent distortion of its physical structure, allowing for a greater outward permeation of cytoplasmic components. Xu et al. [22] observed that, aside from the previously mentioned physical damage, the active compounds in clove buds’ essential oil also inhibit the synthesis of DNA and proteins that are vital for bacterial growth. An in-depth study on the effects of clove essential oil on the growth of *Listeria monocytogenes* [23] demonstrated that the oil presented an inhibitory effect on the expression of β-galactosides and alkaline phosphatase and effectively inhibited respiratory metabolism by the tricarboxylic acid cycle pathway. Bai et al. [24] verified that clove essential oil induced the production of reactive oxygen species and increased the activities of antioxidant enzymes in *S. aureus* cells, leading to cell death due to excessive oxidative stress.

#### 3.1.4. Water Vapor Permeability

Films prepared with 2 g of clove essential oil were tested for water vapor permeability. Control films, consisting solely of cassava waste starch and glycerol (i.e., without the incorporation of essential oil), were also tested for their water vapor permeability to verify the effects of adding clove essential oil on this film property. The results for water vapor permeability of all the films are presented in Table 3. The lower water vapor permeability of the films containing clove essential oil compared to those without it is justified by the fact that clove essential oil mostly comprises hydrophobic compounds, and these are embedded in the film matrix precluding water vapor mobility and interactions with the film molecular network. The values obtained for the WVP of clove oil-incorporated films are similar to those obtained by Fronza et al. [1] for conjugated films of cassava waste starch and locust bean galactomannan (WVP = 0.15 ± 0.01 g mm/m^2^ h kPa). Souza et al. [25] developed films based on cassava starch with added cinnamon and clove oils and observed an increase in water vapor permeability, from 0.41 to 0.62 g mm/m^2^ h kPa, with an increase in cinnamon oil content. Starch extracted from intact bitter cassava was used as precursor material for the preparation of biopolymeric films by Tumwesigye et al. [26], and their average WVP was 0.18 g mm/m^2^ h kPa. It was observed that WVP decreased with increased drying temperatures and increased with increased glycerol content. The increase in WVP by increasing glycerol content was justified by its ability to lower intermolecular forces between the film’s polymer chains, thus facilitating water mobility within the polymer network. No explanation was given for the effects of increasing drying temperature on WVP. Luchese et al. [27] investigated the effects of the amount of starch used to prepare films on their respective properties. Corn (*Zea mays* L.) and cassava (*Manihot esculenta* L.) starch-based films were manufactured by casting, with increasing starch contents varying from 20 to 60 g kg^−1^. Principal Component Analysis results demonstrated that increasing starch contents led to increases in films’ thicknesses, from 0.07 to 0.17 mm, and reductions in films’ water vapor permeabilities, from 0.42 to 0.15 g mm h^−1^ m^−2^ kPa^−1^.

Cassava starch films were developed by dos Santos Caetano et al. [28] with the addition of oregano essential oil, and the water vapor permeability of the films varied from 0.36 to 0.66 g mm/m^2^ h kPa. Results demonstrated that the addition of oregano oil to the films promoted a decrease in water vapor permeability even when the glycerol amount was increased. These results were justified by the fact that, although the addition of glycerol favors an increase in the films’ WVP, the highly hydrophobic compounds of the added essential oil were able to counteract such action. Kang and Song [29] evaluated the physical properties of Job’s tears starch (*Coix lachryma-jobi* L.) films incorporated with clove bud essential oil and determined that films’ water vapor permeability increased with increasing contents of essential oil. This trend was attributed to the increased formation of holes within the film structure.

Al-Hashimi et al. [30] prepared films based on Millet starch with incorporated clove essential oil and observed that the films’ WVP increased from 0.28 to 0.52 g mm/m^2^ h kPa as the contents of clove oil in the films were increased from 0% (control) to 3%. In this case, the added amount of clove oil was not able to counteract the facilitated water mobility action of the incorporated hydrophilic glycerol. Bangar et al. [31] studied the effects of incorporating kudzu cellulose nanocrystals (CNCs), stabilized by the pickering emulsion of clove bud oil, into pearl millet starch films. The reinforced film presented a reduced water vapor permeability (0.2 g mm/m^2^ h kPa) when compared to that of the film without reinforcement (0.27 g mm/m^2^ h kPa). The observed change in WVP was attributed to the increased tortuosity of the diffusion path produced by the well-dispersed CNCs and essential oil compounds in the film matrix, causing hindrance in water mobility through the film polymer network. Corn starch-based composite films incorporated with clove essential oil nanoemulsion were successfully prepared by Fan et al. [32]. The physical and chemical properties of the composite films were investigated at various contents of essential oil nanoemulsion. Increasing the oil nanoemulsion contents in the films led to significantly decreased water vapor permeabilities, attributed to the hydrophobicity of the compounds in the oil embedded in the film matrix. Arias et al. [33] employed a central composite rotatable design to optimize the formulation of potato and cassava starch-based films and determined the respective properties. Water vapor permeabilities varied from 0.1 to 0.33 g mm/m^2^ h kPa for films based on cassava starch and from 0.11 to 0.28 g mm/m^2^ h kPa for films based on potato starch.

It can be clearly observed that the values for WVP obtained for the films in our study are at the lower end of the range of WVP values published in the literature for films comprising starch with incorporated essential oils. Although the determined values of WVP are about 10 times higher than those for films based on petroleum-derived polymers, they are sufficiently low to allow for the application of the prepared films for packaging fresh food for a short period of time.

#### 3.1.5. Mechanical Properties

The results obtained for mechanical properties of the prepared films are presented in Table 4. The standard deviations for both tensile strength and elongation at break are relatively high, justified by the non-homogeneous nature of the cassava waste extracts and the consequent heterogeneity of the composition of the films. In the case of the films without cassava periderm, the incorporation of essential oil led to a decrease in tensile strength. The tensile strengths of the films with cassava periderm, with and without incorporated essential oil, were statistically similar.

Souza et al. [25] added cinnamon essential oil to cassava starch films and determined that the incorporation of the essential oil and emulsifier into the films caused a significant reduction in tensile strength, from 3.75 ± 0.70 to 2.32 ± 0.40 MPa, with increasing elongation at break from 128.81 ± 18.67 to 256.13 ± 48.57%, with the variations justified by the reduction of intermolecular interactions between polymeric chains caused by both the essential oil and the emulsifier. Tumwesigye et al. [26] prepared films using starch extracted from intact bitter cassava as precursor material and determined the optimal formulation considering the effects of glycerol content and drying temperature on the resultant films. It was observed that increasing the amount of glycerol in the film caused a reduction in tensile strength and an increase in elongation at break and that increasing the drying temperature caused a decrease in both tensile strength and elongation at break.

Luchese et al. [27] investigated the effects of varying the amounts of both corn and cassava starches used to prepare films on their respective properties and observed that an increase in the starch content promoted increases in the tensile strength and elongation at break of the starch-based films. Tensile strengths varied from 1.6 to 8.8 MPa, and elongations at break varied from 21 to 124%. Cassava starch films were developed by Caetano et al. [28] with the addition of oregano essential oil, and the results for mechanical properties of the films demonstrated that the incorporation of essential oil had a negative impact on the tensile strength whereas it caused a positive impact on the elongation at break. The tensile strengths of the studied formulations varied from 0.32 to 1.07 MPa, and elongations at break varied from 114.04 to 258.84%. Kang and Song [29] evaluated the mechanical properties of Job’s tears starch films incorporated with clove bud essential oil and determined that increases in the essential oil content of the films led to decreases in tensile strength (from 13.13 to 8.58 MPa) and increases in elongation at break (from 20.56 to 41.3%). The variations in tensile strength and elongation at break were attributed to a partial replacement of intermolecular starch–starch interactions by intermolecular starch–essential oil interactions in the film matrix, resulting in a diminished rigidity of the film polymeric network and increased film flexibility.

Al-Hashimi et al. [30] prepared films based on millet starch with incorporated clove essential oil and observed that the films’ tensile strength decreased from 10.52 MPa for the control film (no essential oil incorporated) to 6.25 MPa for the film with 3% essential oil, as the oil content was increased by increments of 1%. The elongation at break of the studied films also presented a decrease in values as the amount of essential oil in the films was increased and was in the range of 9.3% for the control film to 5.67% for the film with 3% oil. The effects of incorporating kudzu cellulose nanocrystals emulsified with clove bud oil into pearl millet starch films were studied by Bangar et al. [31], and the reinforced film presented an increased tensile strength (from 3.9 to 16.7 MPa) and a decreased elongation at break (from 54.2 to 30%) regarding the control film.

The mechanical properties of corn starch-based composite films incorporated with clove essential oil nanoemulsion were investigated by Fan et al. [32] at various contents of essential oil nanoemulsion. The tensile strength of the films increased significantly, while the elongation at break decreased with increasing contents of essential oil nanoemulsion of the films. The increase in tensile strength and reduction in elongation at break were attributed to the decrease in the free volume and molecular fluidity of the starch polymer caused by a strong interaction between the essential oil and the film polymeric matrix. Arias et al. [33] performed an optimization study of the formulation of films based on starch from cassava and potato and determined the films’ tensile strengths to decrease and the elongation at break to increase as the incorporated amount of the plasticizer sorbitol was increased. The results obtained in the present study for mechanical properties of the prepared films are at par with the results published in the literature for mechanical properties of starch-based films with or without the incorporation of essential oils.

It is noteworthy to point out that eugenol has been successfully used as a plasticizer in some studies of biopolymeric films [34,35,36]. To act as a plasticizer, the molecule must be able to disrupt the hydrogen-bond interactions between the polymers in the film matrix and to form hydrogen bonds with these molecules, hence lowering the crystallinity of the biopolymeric matrix and allowing for greater polymeric chain mobility. In our study, however, eugenol was incorporated into the film together with glycerol, the latter being the most widely used plasticizer in biopolymeric films’ studies. Eugenol has a single hydroxyl group that might interact with the biopolymers in the matrix via hydrogen bond, whereas glycerol has three. Glycerol is more polar and more soluble in water than eugenol, and the glycerol molecule is significantly smaller than eugenol’s, thus suffering less steric hinderance for interaction with the biopolymeric matrix than the latter. Therefore, in our study, eugenol’s contribution to the disruption of hydrogen bonds between starch molecules, and further interaction with these starch molecules by hydrogen bonds, is deemed insignificant compared to that of glycerol. The size of the plasticizer molecule was demonstrated by Sothornvit and Krochta [37] to have significant effects on the plasti-cizer’s efficiency; the smaller the molecule sizes the higher the plasticizing efficiency.

## 4. Conclusions

Starch was successfully extracted from cassava waste and used as precursor materials for the preparation of biopolymeric films. Films were prepared with and without the cassava periderm. Essential oil extracted from clove buds was incorporated into the prepared films and tested for antimicrobial activity against *Staphylococcus aureus* CCCB S007, *Salmonella* Typhimurium (CCCB S004) and *Listeria monocytogenes* serotype 4 b (ATCC 19115). All the prepared films were equally effective in preventing the growth of all the tested Gram-positive and Gram-negative microorganisms. The water vapor permeability of all the films was at the lower end of the range published in the literature for starch-based films. The incorporation of clove essential oil into the films caused a reduction in tensile strength when compared to the control films without the incorporation of essential oil. The films’ elongation at break did not vary significantly with the incorporation of essential oil.

The availability of eugenol from renewable sustainable sources and its ability to prevent bacterial growth, together with its low toxicity to humans and characteristic hydrophobicity, make it an ideal candidate for antimicrobial agent to be incorporated into biopolymeric films. The upcycling of cassava processing waste by using its starch content to produce biopolymeric films was demonstrated feasible, allowing for a reduction in the amount of waste generated during processing and fitting into the concept of circular economy, with the possibility of added revenues to the cassava producers. The employment of green chemistry in the preparation of the films, by the exclusive use of water to extract cloves’ essential oil and to extract starch from cassava waste, led to the development of a sustainable green process to produce antimicrobial biopolymeric films. The incorporation of eugenol into cassava waste starch films not only introduced antimicrobial activity to the film but also improved the water vapor permeability without prejudice to the films’ mechanical properties, which were in the lower end of the range of mechanical properties of polyethylene, a non-renewable, non-biodegradable petroleum-derived polymer. Based on these premises, the prepared films are deemed a strong candidate for use as packaging for fresh foods with a short period of time between production and consumption.

## Figures and Tables

**Table 1:** Effect of oxidizing treatment on film color parameters.

**Film**	**Treatment**	**Color Parameters**		
		L*	c*	h*
NCPF	No Treatment	49.03 ± 0.08 c	18.90 ± 0.33 f	72.19 ± 0.27 c
WCPF		36.91 ± 0.07 f	20.54 ± 0.35 f	59.01 ± 0.94 e
NCPF	NaClO 2%	49.12 ± 0.11 c	31.12 ± 0.08 b	72.77 ± 0.59 c
	NaClO 6%	58.50 ± 0.67 a	25.95 ± 0.46 d	80.21 ± 0.11 a
	NaClO 10%	55.66 ± 0.30 b	29.63 ± 0.40 b	77.35 ± 0.25 b
WCPF	NaClO 2%	35.42 ± 0.03 f	23.52 ± 0.15 e	53.86 ± 0.88 f
	NaClO 6%	48.05 ± 0.13 c	38.97 ± 0.18 a	65.32 ± 0.33 d
	NaClO 10%	42.26 ± 0.58 d	37.98 ± 1.52 a	57.30 ± 1.14 e
NCPF	H_2_O_2_ 2%	55.67 ± 1.40 b	27.56 ± 1.60 d	73.34 ± 0.45 c
	H_2_O_2_ 6%	55.13 ± 1.36 b	31.26 ± 0.19 c	73.91 ± 0.13 c
	H_2_O_2_ 10%	57.73 ± 0.51 a	31.93 ± 0.34 c	77.92 ± 0.09 b
WCPF	H_2_O_2_ 2%	40.77 ± 0.15 d	31.58 ± 4.01 c	54.43 ± 0.95 f
	H_2_O_2_ 6%	36.40 ± 0.34 f	35.04 ± 1.22 b	52.71 ± 0.57 f
	H_2_O_2_ 10%	38.55 ± 0.02 e	32.90 ± 0.53 c	51.57 ± 0.73 f

NCPF: film without corky periderm; WCPF: film with corky periderm. Results are expressed as mean ± standard deviation (n = 3). Different letters in the same column indicate that values are statistically different based on Tukey Test (*p* < 0.05).

**Table 2:** Antimicrobial activities of films functionalized with clove extract in comparison to control samples.

Antimicrobial Activity of Functionalized Films		
Film	Bacteria	Inhibition Zone (mm)
NCPF	*Staphylococcus aureus*	11.00 ± 5.04 a
	*Listeria monocytogenes*	11.80 ± 2.96 a
	*Salmonella enterica* Typhimurium	11.72 ± 0.87 a
WCPF	*Staphylococcus aureus*	9.10 ± 5.50 a
	*Listeria monocytogenes*	10.57 ± 3.28 a
	*Salmonella enterica* Typhimurium	11.87 ± 1.62 a
Eugenol	*Staphylococcus aureus*	23.75 ± 3.37 a
	*Listeria monocytogenes*	*
	*Salmonella enterica* Typhimurium	*
Clove essential oil	*Staphylococcus aureus*	19.37 ± 2.68 a
	*Listeria monocytogenes*	*
	*Salmonella enterica* Typhimurium	22.50 ± 2.05 a

NCPF: film without corky periderm; WCPF: film with corky periderm. Both NCPF and WCPF were treated with 6% NaClO. Results are expressed as mean ± standard deviation (n = 3). Different letters in the same column indicate that values are statistically different based on Tukey Test (*p* < 0.05). * Values not determined due to complete growth inhibition.

**Table 3:** Thickness and water vapor permeability of the prepared films, with and without incorporated clove essential oil.

Film	Thickness (mm)	WVP (g mm/m^2^ h kPa)
NCPF *	0.270 ± 0.06 a	0.14 ± 0.06 b
WCPF *	0.236 ± 0.02 a	0.15 ± 0.03 b
NCPF	0.206 ± 0.06 b	0.23 ± 0.04 a
WCPF	0.207 ± 0.03 b	0.27 ± 0.08 a

* Films containing clove essential oil. NCPF: film without corky periderm; WCPF: film with corky periderm. Both NCPF and WCPF were treated with 6% NaClO. Results are expressed as mean ± standard deviation (n = 5). Different letters in the same column indicate that values are statistically different based on Tukey Test (*p* < 0.05).

**Table 4:** Mechanical properties of the prepared films, with and without incorporated clove essential oil.

Film	Tensile Strength (MPa)	Elongation at Break (%)
NCPF *	1.7 ± 0.4 b	32.90 ± 14.31 a
WCPF *	1.5 ± 0.4 b	48.01 ± 18.77 a
NCPF	3.2 ± 0.3 a	21.40 ± 13.0 a
WCPF	2.1 ± 0.4 a,b	40.24 ± 11.38 a

* Films containing clove essential oil. NCPF: film without corky periderm; WCPF: film with corky periderm. Both NCPF and WCPF were treated with 6% NaClO. Results are expressed as mean ± standard deviation (n = 6). Different letters in the same column indicate that values are statistically different based on Tukey Test (*p* < 0.05).

## Data Availability

The original contributions presented in the study are included in the article, further inquiries can be directed at the corresponding author.
